# Increased Circulating Levels of Alpha-Ketoglutarate in Morbidly Obese Women with Non-Alcoholic Fatty Liver Disease

**DOI:** 10.1371/journal.pone.0154601

**Published:** 2016-04-28

**Authors:** Gemma Aragonès, Teresa Auguet, Alba Berlanga, Esther Guiu-Jurado, Salomé Martinez, Sandra Armengol, Fàtima Sabench, Rosa Ras, Mercè Hernandez, Carmen Aguilar, Josep Colom, Joan Josep Sirvent, Daniel Del Castillo, Cristóbal Richart

**Affiliations:** 1 Grup de Recerca GEMMAIR (AGAUR)- Medicina Aplicada. Departament de Medicina i Cirurgia. Universitat Rovira i Virgili (URV), Institut d’Investigació Sanitària Pere Virgili (IISPV), 43007, Tarragona, Spain; 2 Servei Medicina Interna, Hospital Universitari Joan XXIII Tarragona. Mallafré Guasch, 4, 43007, Tarragona, Spain; 3 Servei Anatomia Patològica, Hospital Universitari Joan XXIII Tarragona. Mallafré Guasch, 4, 43007, Tarragona, Spain; 4 Servei de Cirurgia. Hospital Sant Joan de Reus. Departament de Medicina i Cirurgia. Universitat Rovira i Virgili (URV), IISPV, Avinguda Doctor Josep Laporte, 2, 43204, Reus, Spain; 5 Group of Research on Omic Methodologies (GROM). Centre for Omic Sciences (COS), Reus, Spain; 6 Servei Medicina Interna, Hospital Sant Joan de Reus. Departament de Medicina i Cirurgia. Universitat Rovira i Virgili (URV), IISPV, Avinguda Doctor Josep Laporte, 2, 43204, Reus, Spain; RWTH Aachen, GERMANY

## Abstract

**Background:**

Non-alcoholic fatty liver disease (NAFLD) causes a wide spectrum of liver damage, ranging from simple steatosis to cirrhosis. However, simple steatosis (SS) and steatohepatitis (NASH) cannot yet be distinguished by clinical or laboratory features. The aim of this study was to assess the relationship between alpha-ketoglutarate and the degrees of NAFLD in morbidly obese patients.

**Materials and Methods:**

We used a gas chromatography-quadruple time-of-flight-mass spectrometry analysis to quantify alpha-ketoglutarate in serum from normal-weight subjects (n = 30) and morbidly obese women (n = 97) with or without NAFLD.

**Results:**

We found that serum levels of alpha-ketoglutarate were significantly higher in morbidly obese women than in normal-weight women. We showed that circulating levels of alpha-ketoglutarate were lower in lean controls and morbidly obese patients without NAFLD. We also found that alpha-ketoglutarate serum levels were higher in both SS and NASH than in normal liver of morbidly obese patients. However, there was no difference between SS and NASH. Moreover, we observed that circulating levels of alpha-ketoglutarate were associated with glucose metabolism parameters, lipid profile, hepatic enzymes and steatosis degree. In addition, diagnostic performance of alpha-ketoglutarate has been analyzed in NAFLD patients. The AUROC curves from patients with liver steatosis exhibited an acceptable clinical utility. Finally, we showed that the combination of biomarkers (AST, ALT and alpha-ketoglutarate) had the highest accuracy in diagnosing liver steatosis.

**Conclusion:**

These findings suggest that alpha-ketoglutarate can determine the presence of non-alcoholic fatty liver in morbidly obese patients but it is not valid a biomarker for NASH.

## Introduction

Obesity is an important risk factor associated with the metabolic alterations of such diseases as diabetes mellitus type 2, insulin resistance, hyperlipidemia, and non-alcoholic fatty liver disease (NAFLD) [1]. Although obesity is an important risk factor, not all patients with NAFLD are obese [2]. NAFLD has become the most common liver disorder in the developed countries, affecting over one-third of the population [3,4]. The disease causes a wide spectrum of liver damage, ranging from simple steatosis (SS) to cirrhosis. In most cases, SS does not develop into a more severe disease, but ~20–30% of patients have histological signs of fibrosis and necroinflammation, which indicates the presence of non-alcoholic steatohepatitis (NASH) [5]. However, the factors that convert some fatty livers into livers with steatohepatitis are not fully understood.

Alpha-ketoglutarate is a major intermediate metabolite of the tricarboxylic acid cycle and plays an important role in regulating energy metabolism [6]. It is also one of the 12 major precursors for the synthesis of most biochemical substances [7]. Recent findings using metabolomic approaches to investigate alterations in many diseases showed differences in such constituents of the Krebs cycle as alpha-ketoglutarate [8,9]. In this context, a recent study reveals that alpha-ketoglutarate could act as a predictor of morbid obesity-associated non-alcoholic fatty liver disease [10]. In fact, non-invasive methods such as liver ultrasound are already able to diagnose simple steatosis in morbidly obese subjects. However, simple steatosis and NASH can only be distinguished by liver histology and at present cannot be predicted by clinical or laboratory features.

The purpose of this study was to analyse the relationship between alpha-ketoglutarate and non-alcoholic fatty liver disease in morbidly obese patients and to assess whether the serum concentration of alpha-ketoglutarate is associated with the severity of disease and indicates a diagnosis of non-alcoholic steatohepatitis.

## Materials and Methods

### General Protocol

The study was approved by the institutional review board “Comitè d’Ètica d’Investigació Clínica, Hospital Universitari de Tarragona Joan XXIII” (23c/2015). All participants gave written informed consent for participation in medical research. We included 127 Spanish women of Western European descent: 30 normal-weight controls (Body mass index (BMI) < 25 kg/m^2^) and 97 morbidly obese (MO) (BMI > 40 kg/m^2^). Liver biopsies from MO were obtained during planned laparoscopic bariatric surgery. All biopsies were performed for clinical indications. The diagnosis of NAFLD was made on the basis of the following criteria: liver pathology, an intake of less than 10 gr. of ethanol/day, and exclusion of other liver diseases.

The weight of all subjects in the population studied was stable with no fluctuation greater than 2% of body weight for at least 3 months. The exclusion criteria for morbidly obese patients were: (1) concurrent use of medications known to produce hepatic steatosis, (2) diabetic women that were receiving insulin or on medication likely to influence endogenous insulin levels, (3) patients who had an acute illness, current evidence of acute or chronic inflammatory or infectious diseases or end-stage malignant diseases.

Liver samples were scored by experienced hepatopathologists using the methods described elsewhere [11]. According to their liver pathology, MO patients were sub-classified into the following groups: normal liver (NL) histology (n = 18), simple steatosis (SS) (micro/macrovesicular steatosis without inflammation or fibrosis, n = 41) and non-alcoholic steatohepatitis (NASH) (n = 38).

Each patient was subject to a complete anthropometrical, biochemical and physical examination.

### Quantitative Analysis of Alpha-Ketoglutarate

Aplha-ketoglutarate was quantitatively determined in serum samples by GC/MS at the Centre for Omic Sciences (Rovira i Virgili University). The methodology used has been reported elsewhere [10]. Samples were analysed in a 7890A Series gas chromatograph coupled to a 7200 GCqTOF MS (Agilent Technologies, Santa Clara, U.S.A.). The chromatographic column was a J&W Scientific HP5-MS (30 m x 0.25 mm i.d., 0.25 μm film) (Agilent Technologies). The calibration curve showed good linearity in the studied range of 0.13 to 10 mg/L, with a determination coefficient (*R*^2^) of 0.9993. The extraction recovery was 99.7%, while accuracy was 101.7%. The intraday and interday precision had a relative standard deviation of, respectively, 4.1% and 4.8% (RSD, *n* = 3). MDL was 0.05 mg/L, while MQL was 0.13 mg/L.

### Statistical Analysis

All the values reported were analyzed using the SPSS/PC+ for windows statistical package (version 22.0; SPSS, Chicago, IL). One way ANOVA with post-hoc Turkey test was used to compare continuous variables between groups. The strength of association between variables was calculated using Pearson’s method and Spearman’s ρ-correlation test. ROC curves were used to evaluate performance as a biomarker of circulating alpha-ketoglutarate levels in the diagnosis of NAFLD and NASH. *P-*values<0.05 were considered to be statistically significant.

## Results

### Baseline Characteristics of Subjects

The general characteristics, biochemical measurements and histopathological characteristics of the population studied are shown in Table 1. First, we classified the patients into two groups according to their BMI: normal-weight subjects (BMI < 25 kg/m^2^; n = 30), and morbidly obese women (MO; BMI > 40 kg/m^2^; n = 97). The two groups were comparable in terms of age (*p* = 0.204). As expected, biochemical analyses indicated that MO patients had significantly higher levels of fasting glucose, insulin, HOMA2-IR, HbA1c, systolic blood pressure (SBP), diastolic blood pressure (DBP) and lipid profile (*p*<0.05) than normal-weight subjects. HDL-C was significantly lower in the morbidly obese patients than in the normal-weight group (*p*<0.001). Our results also indicated that AST, ALT and ALP activity was higher in the morbidly obese cohort (*p*<0.05).

**Table 1:** Anthropometric and metabolic variables of study cohort classified according the BMI and histopathological characteristics.

	NORMAL-WEIGHT (N = 30)	MORBID OBESITY (N = 97)			
Variables	Mean + SD	Mean + SD			
	-	NL (N = 18)	NAFLD (N = 79)	SS (n = 41)	NASH (N = 38)
Age (years)	44.12±7.56	43.95±10.26	47.07±8.89	46.13±9.44	48.14±8.21
Weight (kg)	58.20±7.76*	111.98±20.89	120.29±16.81	122.20±16.54	118.13±17.07
WC (cm)	76.10±9.20*	121.78±22.85	128.69±9.86	128.15±8.58	129.26±11.27
BMI (kg/m^2^)	22.83±1.92*	44.90±9.11	47.19±6.33	47.88±7.10	46.41±5.31
SBP (mmHg)	118.33±11.23*	128.00±14.47	132.92±17.35	130.03±16.16	135.74±18.21
DBP (mmHg)	69.08±9.20*	74.26±8.47	75.32±14.12	70.81±12.53	79.71±14.34^**#**^
Glucose (mg/dL)	85.68±14.90*	86.17±14.01	125.66±39.27^**§**^	122.31±37.56	129.37±41.27
Insulin (mUI/L)	7.26±4.74*	12.57±8.15	19.64±13.98^**§**^	18.51±13.33	20.89±14.74
HbA1c (%)	4.83±0.26*	4.99±0.47	6.14±1.38^**§**^	6.15±1.28	6.12±1.49
HOMA2-IR	0.98±0.68*	1.50±1.06	2.53±1.67^**§**^	2.25±1.04	2.66±1.89
Triglycerides (mg/dL)	90.32±55.06*	135.26±66.95	175.44±93.13^**§**^	174.50±101.53	176.47±84.22
Cholesterol (mg/dl)	192.05±28.68	165.49±29.95	175.89±33.00	167.07±33.42	185.65±30.04^**#**^
HDL-C (mg/dL)	58.64±13.41*	48.62±13.44	39.33±9.92^**§**^	37.63±10.63	41.18±8.86
LDL-C (mg/dL)	114.64±25.42*	88.48±25.16	103.73±26.96^**§**^	97.69±26.48	111.05±26.07^**#**^
AST (U/L)	21.33±9.85*	21.86±6.09	46.26±32.51^**§**^	48.34±35.33	43.99±29.50
ALT (U/L)	18.42±8.24*	20.76±8.18	43.33±29.14^**§**^	47.73±30.93	44.78±27.37
GGT (U/L)	22.92±37.30	20.00±21.61	38.73±41.37	35.36±38.96	42.10±43.93
ALP (U/L)	59.00±21.12*	60.64±11.41	72.10±23.35^**§**^	69.79±21.80	74.73±25.04
***Liver histological features***					
Steatosis grade 1/2/3	-	-	30/31/18	10/20/11	20/11/7
Lobular inflammatory grade 0/1/2/3	-	-	40/23/14/2	40/1/0/0	0/22/14/2^**#**^
Hepatocellular ballooning 0/1/2	-	-	40/35/4	40/1/0	0/34/4^**#**^
NAFLD activity score	-	-	3.08±1.51	1.98±0.82	4.30±1.10^**#**^
Fibrosis Staging 0/1/2/3/4	-	-	65/9/1/1/3	39/0/1/0/1	26/9/0/1/2

NL, normal liver; NAFLD, non-alcoholic fatty liver disease; SS, simple steatosis; NASH, non-alcoholic steatohepatitis; WC, waist circumference; BMI, body mass index; SBP, systolic blood pressure; DBP, diastolic blood pressure; HbA1c, glycosylated hemoglobin; HOMA2-IR, homeostatic model assessment method-insulin resistance, HDL-C, high density lipoprotein cholesterol; LDL-C, low density lipoprotein cholesterol; AST, aspartate aminotransferase; ALT, alanine aminotransferase; GGT, gamma-glutamyltransferase; ALP, alkaline phosphatase. Data are expressed as the mean ± SD.

*Significant differences between normal weight controls and morbidly obese group (*P* < 0.05).

^**§**^Significant differences between morbidly obese women without NAFLD and morbidly obese women with NAFLD (*P* < 0.05).

^**#**^Significant differences between SS and NASH (*P* < 0.05). Insulin resistance was estimated using homeostasis model assessment of IR (HOMA2-IR) [27].

Secondly, we classified the morbidly obese cohort according to the liver pathology into normal liver (NL, n = 18) and non-alcoholic fatty liver disease (NAFLD, N = 79). Our results indicated that fasting glucose, insulin, HOMA2-IR, HbA1c, triglycerides and LDL-C levels were significantly higher in NAFLD group than in NL group (*p*<0.05). Also, HDL-C was significantly lower in the morbidly obese patients with NAFLD (*p* = 0.001). Regarding hepatic enzymes, AST, ALT and ALP activity was higher in NAFLD cohort (*p*<0.05).

Finally, we divided morbidly obese cohort with NAFLD according to the liver pathology into simple steatosis (SS, n = 41) and non-alcoholic steatohepatitis (NASH, n = 38). We found that DBP, cholesterol and LDL-C levels were significantly higher in NASH group than in SS patients (*p*<0.05).

### Serum Levels of Alpha-Ketoglutarate

**Fig 1:**
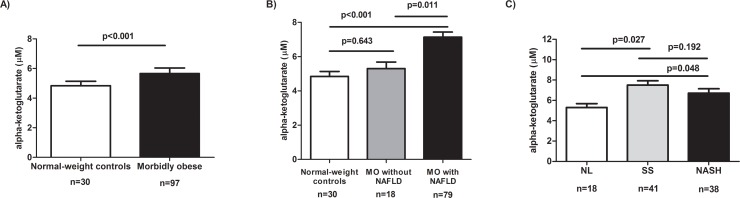
Circulating levels of alpha-ketoglutarate in study cohort. Circulating levels of alpha-ketoglutarate in normal-weight controls and morbidly obese women (**A**); in normal-weight controls, morbidly obese women (MO) without non-alcoholic fatty disease (NAFLD) and morbidly obese women (MO) with NAFLD (**B**); and in morbidly obese women according to the liver pathology (**C**). NL, morbidly obese women with normal liver; SS, morbidly obese women with simple steatosis; NASH, morbidly obese women with steatohepatitis. Results are shown as mean ± SD. *p*< 0.05 are considered statistically significant.

We analysed the circulating levels of alpha-ketoglutarate in both normal-weight subjects and MO. We found that serum levels of alpha-ketoglutarate were significantly higher in MO than in normal-weight subjects (*p*<0.001, Fig 1A).

When we analysed the circulating levels of alpha-ketoglutarate respect to NAFLD presence, it is important to note that there was no significant difference between normal-weight subjects and morbidly obese women without NAFLD (*p =* 0.643, Fig 1B). Moreover, serum alpha-ketoglutarate levels were significantly higher in MO with NAFLD than MO without NAFLD and normal-weight subjects (*p =* 0.011 and *p*<0.001, respectively, Fig 1B).

In order to study the possible role of alpha-ketoglutarate in NAFLD, we classified the cohort of morbidly obese women according to liver pathology. We found that alphaketoglutarate serum levels were higher in both SS and NASH than in NL (*p* = 0.027 and *p* = 0.048, respectively, Fig 1C). However, there were no differences between SS and NASH (*p* = 0.192).

### Correlations between the Levels of Alpha-Ketoglutarate and Biochemical Variables and Histopathological Parameters

**Fig 2:**
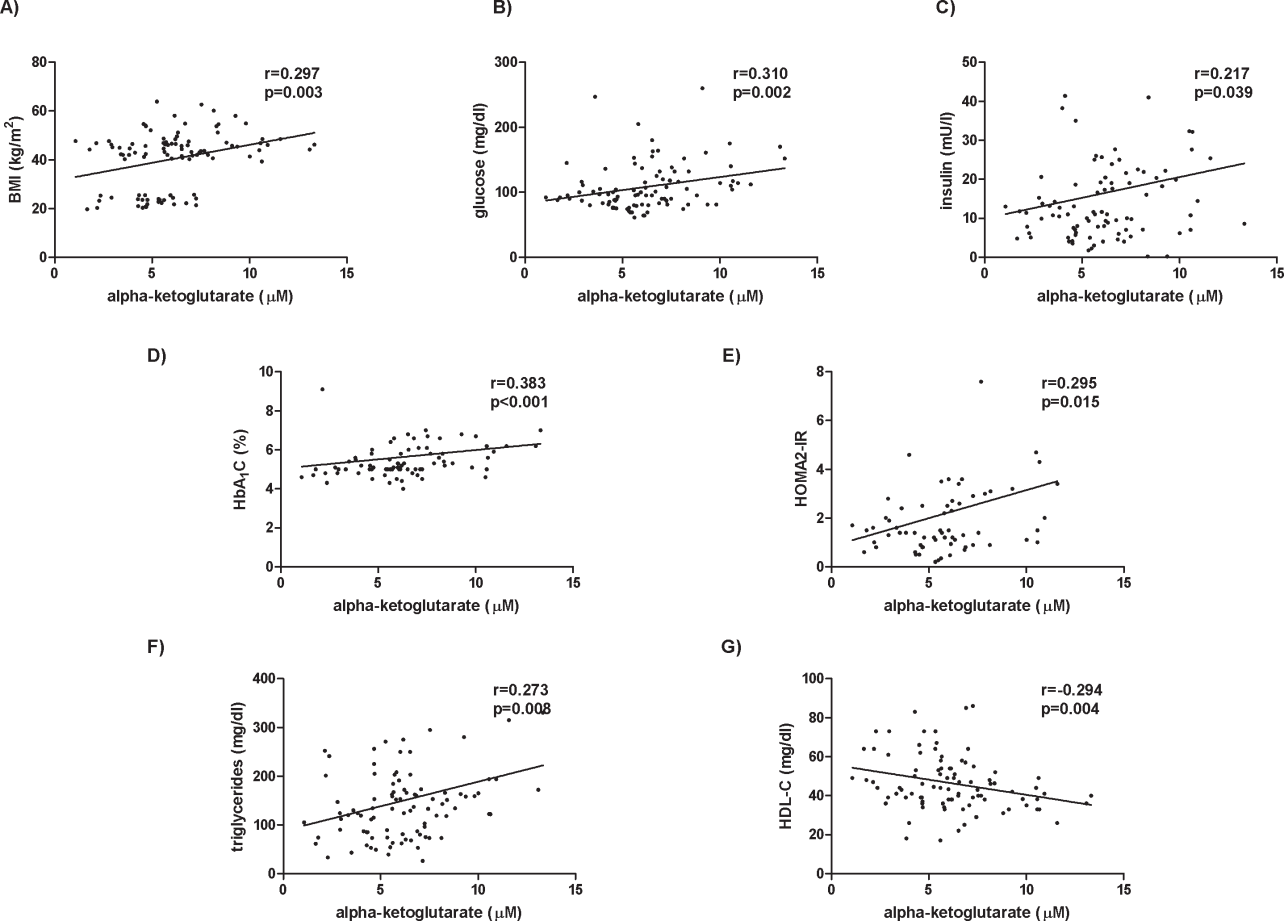
Significant correlations between alpha-ketoglutarate levels and anthropometrical and biochemical variables. The strength of association between variables was calculated using Spearman’s *r* correlation test. BMI, body mass index; HbA1c, glycosylated hemoglobin; HOMA2-IR, homeostatic model assessment method-insulin resistance, HDL-C, high density lipoprotein cholesterol.

Alpha-ketoglutarate circulating levels correlated positively with BMI (r = 0.321; *p*<0.001, Fig 2A), glucose metabolism parameters (glucose: r = 0.323; *p*<0.001; insulin: r = 0.201; *p* = 0.033; HbA1c: r = 0.466; *p*<0.001; HOMA2-IR: r = 0.268; *p* = 0.024; Fig 2B–2E), triglycerides (r = 0.263; *p* = 0.004; Fig 2F) and inversely with HDL-C (r = -0.311; *p* = 0.001; Fig 2G). Moreover, we found that circulating levels of alpha-ketoglutarate were significantly associated with type 2 diabetes (r = 0.296, *p* = 0.001) and dyslipidemia (r = 0.195, *p* = 0.036).

In addition, Table 2 showed the correlations between alpha-ketoglutarate and hepatic parameters. Alpha-ketoglutarate levels correlated with hepatic enzymes (AST: r = 0.382; *p*<0.001; ALT: r = 0.442; *p*<0.001; GGT: r = 0.462; *p*<0.001; ALP: r = 0.240; *p* = 0.011). Regarding histopathological features, alpha-ketoglutarate only correlated with steatosis degree (r = 0.275; p = 0.007, Table 2).

**Table 2:** Correlations between the levels of alpha-ketoglutarate and hepatic enzymes and histopathological parameters in the population studied.

Variables	Alpha-ketoglutarate	
	r	p-value
AST (U/l)	0.382	<0.001
ALT (U/l)	0.442	<0.001
GGT (U/l)	0.462	<0.001
ALP (U/l)	0.240	0.011
Steatosis grade	0.275	0.007
Lobular inflammation	-0.076	0.466
Ballooning	-0.019	0.857
NAFLD activity score	0.091	0.382
Fibrosis	0.096	0.350

AST, aspartate aminotransferase; ALT, alanine aminotransferase; GGT, gamma-glutamyltransferase; ALP, alkaline phosphatase; NAFLD, non-alcoholic fatty liver disease. The strength of association between variables was calculated using Spearman’s *r* correlation test. *P*<0.05 is considered statistically significant.

### Serum Alpha-Ketoglutarate as a Biomarker

**Fig 3:**
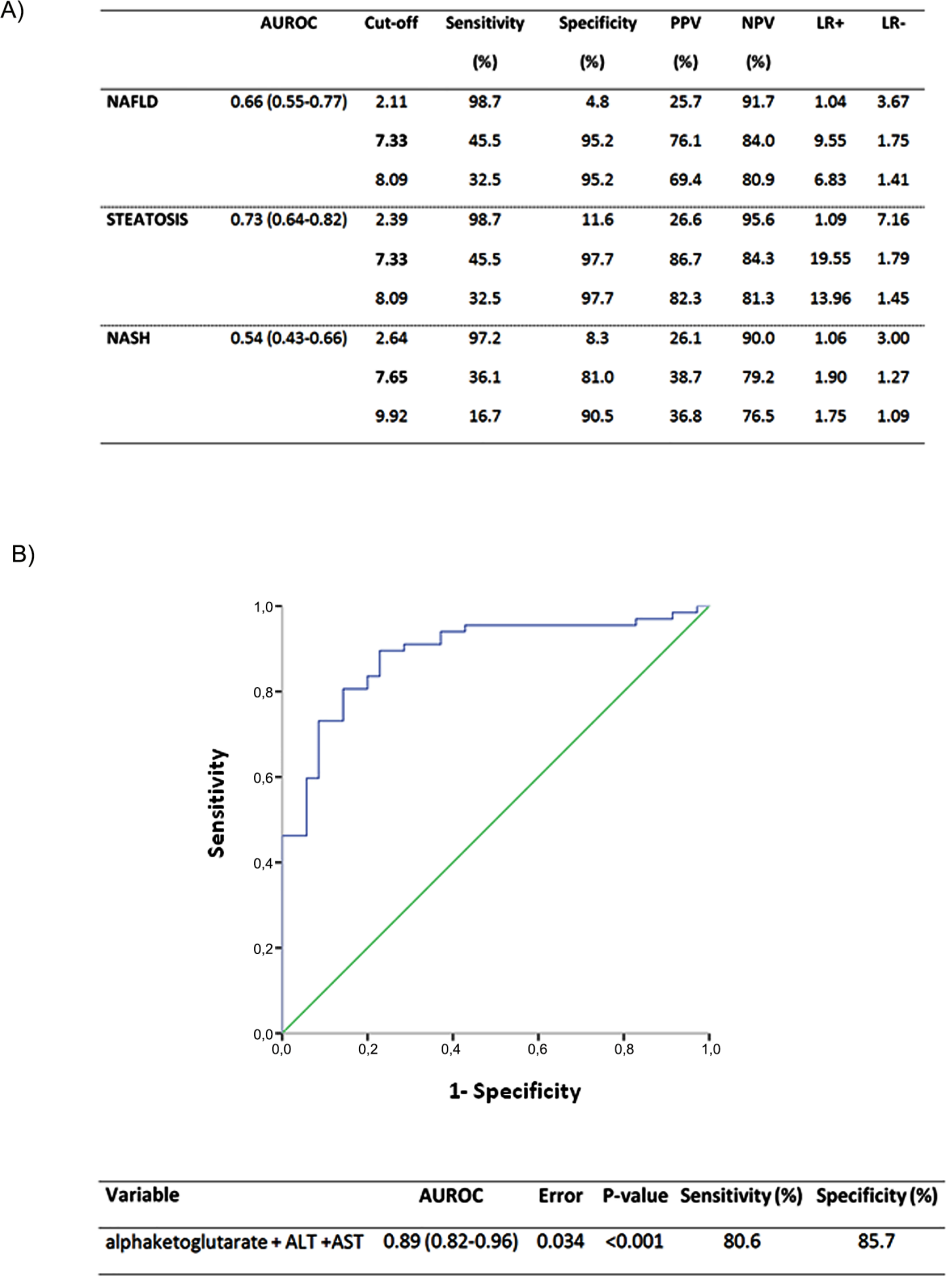
Serum alpha-ketoglutarate as a biomarker. (**A**) Accuracy of alpha-ketoglutarate biomarker in population studied. Represent the performance for discriminating NAFLD, steatosis and NASH from non- diseased patients. The three values of optimum cutting were selected based on obtaining a first cutoff of high sensitivity (>90%), a second cutoff that included the best combination of sensitivity and specificity according to the Youden index and a third, which prioritized specificity (>90%) (**B**) Evaluation of a multimetabolite model as biomarkers of NAFLD by the receiver operator characteristic (AUROC) curves. AUROC, area under the curve of receiver operating characteristics; ALT, alanine aminotransferase; AST, aspartate aminotransferase; LR+, positive likelihood ratio; LR-, negative likelihood ratio; NAFLD, non-alcoholic fatty liver disease; NASH, non-alcoholic steatohepatitis; NPV, negative prespective value; PPV, positive prespective value.

As a final step, diagnostic performance of alpha-ketoglutarate has been analyzed in NAFLD patients determining a cutoff point and area under the curve to establish the diagnosis of this entity (Fig 3A). On one hand, the accuracy of alpha-ketoglutarate to discriminate NAFLD from non-NAFLD subjects showed on average an area under the ROC (AUROC) curve of about 0.66 (0.55–0.77) and to discriminate an advanced disease (NASH) from a mild clinical form showed an AUROC curve of about 0.54 (0.43–0.66; Fig 3A). On the other hand, we found that alpha-ketoglutarate had higher accuracy in detecting liver steatosis (0.76 (0.64–0.82); Fig 3A). Thus, the AUROC curves from patients with liver steatosis exhibited an acceptable clinical utility; however we could observe that alpha-ketoglutarate is not a valid biomarker for diagnosing NASH.

In addition, we have evaluated a multimetabolite model including ALT, AST and alpha-ketoglutarate, as biomarkers of NAFLD, by the receiver operator characteristic curves (Fig 3B). We observed that this combination of biomarkers had the highest accuracy in diagnosing liver steatosis with AUROC of about 0.89 (0.82–0.96).

## Discussion

The present study investigated the potential role of alpha-ketoglutarate as a biomarker of NAFLD disease. The main findings indicate that, first, circulating levels of alpha-ketoglutarate were increased in morbidly obese women. Second, there was no difference between lean controls and morbidly obese patients without NAFLD. However, circulating levels were increased in morbidly obese patients with NAFLD. In obese patients, we showed that most changes in serum levels take place in SS and NASH rather than in NL. Importantly, we observed that there was no difference between NAFLD stages. Although the accuracy of alpha-ketoglutarate as a biomarker of liver steatosis in morbidly obese patients exhibited an acceptable clinical utility, stepwise combination of biomarkers had the highest accuracy in diagnosing liver steatosis.

Currently, it is not possible to predict the outcome of NAFLD or the progression of disease through routinely used blood biomarkers. In fact, liver biopsy is still the gold standard for diagnosing and staging NAFLD, but it is invasive, and has accessibility and reproducibility limitations [12–14]. It would be important to search for non-invasive biomarkers to distinguish NASH from simple steatosis. For this reason, the metabolomic profiling of various liver diseases is increasingly being used in an attempt to find a way of identifying disease biomarkers [15,16]. In this regard, alpha-ketoglutarate has been studied in several diseases [8,9]. As far as obesity is concerned, it is known that alpha-ketoglutarate is related to the fat-mass-and-obesity-associated gene (FTO), and that it is associated with BMI and the regulation of energy homeostasis [17]. In this regard, our findings showed that alpha-ketoglutarate was positively associated with BMI and that it was increased in obese patients. With respect to type 2 diabetes and dyslipidemia associations observed in our study, recently, Ho *et al* have demonstrated novel alterations in multiple intermediates of the citric acid cycle, including alpha-ketoglutarate, which are directly associated with BMI, abdominal obesity, HOMA-IR and triglycerides [18]. Also, reduced alpha-ketoglutarate has been showed after weight loss in obese insulin-resistant patients [19]. Moreover, the citric acid cycle enzyme NADP+-dependent isocitrate dehydrogenase that produces NADPH by converting isocitrate to alpha-ketoglutarate, is expressed in liver and adipocytes, playing a critical role in adipogenesis, specifically regarding to triglycerides and cholesterol [20].

With respect to alpha-ketoglutarate levels in NAFLD patients, we found that this metabolite was increased in morbidly obese patients with NAFLD. In this context, in a metabolomic liver tissue analysis, Clarke *et al*. [16] demonstrated that alpha-ketoglutarate expression was decreased in NASH. Also, the authors compared their results with two previous studies performed in plasma of hepatocarcinoma (HCC) patients. Chen *et al*. and Patterson *et al*. have described increased plasma levels of this metabolite in HCC patients [15,21]. As currently is lacking regarding significance of this metabolite in the pathogenesis of NAFLD or HCC, Clarke *et al*. suggested that the metabolite differences could represent perturbations in specific metabolic pathways that may contribute to the transition from NASH to HCC. The discrepancies with our results might be partially explained by differences in the cohort of the patients studied. Specifically, our work was performed only in serum samples of obese patients with NAFLD (SS or NASH) without HCC. According to our results, a recent study showed that normal-weight controls had lower serum alpha-ketoglutarate levels than obese patients and the measurement of this metabolite differentiated between obese patients with or without NAFLD [10]. In this sense, we also observed that circulating levels of alpha-ketoglutarate were lower in both lean controls and morbidly obese patients without NAFLD. Taking these results together and considering that serum alpha-ketoglutarate levels were increased in MO with NAFLD, our study suggests that this metabolite should be able to determine the presence of NAFLD in obese patients. We also found that there was no significant alteration in alpha-ketoglutarate levels between SS and NASH. Alpha-ketoglutarate, then, seems to be a good biomarker of NAFLD but it does not discriminate between NAFLD stages. This is controversial because previous reports indicate that most changes in gene expression and metabolite levels occur in the transition from steatosis to NASH [22,23].

In order to evaluate the performance of this metabolite in predicting NAFLD in morbidly obese patients, receiver operating characteristic (ROC) curves were obtained. ROC analysis revealed that alpha-ketoglutarate had higher accuracy in detecting liver steatosis in morbidly obese patients. Although this predictive value is not good enough for an ideal biomarker, the performance of alpha-ketoglutarate is quite similar to other reported studies [10].

Our findings suggest that alpha-ketoglutarate can determine the presence of liver steatosis in morbidly obese patients but it is not valid a biomarker for NASH. Nowadays there are no reliable non-invasive biomarkers to distinguish NASH from SS. However, it has been proposed combining scoring systems and imaging methods to improve efficiently diagnose NAFLD/ NASH. In this regard, a number of predictive models to differentiate SS from NASH have been presented [24,25]. In addition, NASH diagnostics uses a combination of CK 18-M30 and M65 levels with adiponectin and resistin values to obtain an AUROC of 0.91 in the test and 0.73 in the validation groups. A recent meta-analysis has analysed the performance of the NashTest® and ActiTest® for the diagnosis of NASH in 494 obese patients and obtained an AUROC of 0.84 for the diagnosis of NASH [26]. Although multiple scoring systems have been developed, further studies are needed to improve the capacity to distinguish NASH from simple steatosis. In this sense, we have evaluated a multimetabolite model including ALT, AST and alpha-ketoglutarate, as biomarkers of NAFLD, by the receiver operator characteristic curves. These results suggest that compared to single biomarker (alpha-ketoglutarate), stepwise combination of different biomarkers can further improve the accuracy in diagnosing liver steatosis.

Our study cohort enabled us to confirm that alpha-ketoglutarate levels were higher in morbidly obese women with NAFLD without the interference of such confounding factors as gender or age. These results cannot be extrapolated to other obesity groups, over-weight women or men.

In conclusion, circulating levels of alpha-ketoglutarate were increased in morbidly obese women with non-alcoholic fatty liver disease. However, we observed that there was no difference between NAFLD stages. Therefore, this metabolite does not discriminate between simple steatosis and steatohepatitis, which suggests that alpha-ketoglutarate is not a valid biomarker for NASH.
